# A better future for injectable contraception?

**DOI:** 10.9745/GHSP-D-14-00158

**Published:** 2014-12-02

## Abstract

- Provision of injectables though drug shops appears practicable and can contribute a marked share of family planning services.
- A potential longer-acting injectable providing at least 6 months of protection appeals to programmatic professionals.
- Subcutaneous administration of DMPA offers major injectable improvements over the current intramuscular approach.
- Ironically, while injectable use will inevitably grow, better choice and wider availability of other methods—especially of long-acting and permanent methods—will reduce injectables′ overall share.

## PLUSES AND MINUSES OF INJECTABLES

Injectables are a leading method in much of the developing world. They have many advantages for the client, the provider, and the system (Table). All the same, the dominant injectable DMPA (depot medroxyprogesterone acetate) in its current intramuscular (IM) form has some pretty important drawbacks—notably, side effects, higher blood levels than needed for most of the 3-month duration, provider bias, possible role in HIV acquisition, and requirement for repeated visits that are a burden to both the system and the client. Despite DMPA's high popularity, continuation rates with DMPA are rather poor. After 1 year of starting DMPA, only about 50% of women, on average, are still using the method—rates similar to those of condom and oral contraceptive users.^1^

**Table:** TABLE. Advantages and Disadvantages of Current Intramuscular DMPA Injectable

**Advantages**	**Disadvantages**
• Highly effective	• Bleeding side effects
• Coitally independent	• Weight gain^a^
• Possible to use without partner's knowledge	• Reduced bone density (temporary)
• Lasts a full 3 months	• Not advised during early breastfeeding by WHO
• 1-month grace period for reinjection	• Delayed return to fertility
• Safe and suitable for nearly all women	• Higher initial blood dosage than needed for efficacy
• Relatively low cost	• Requires repeated visits
• Easy for providers	• System burden (eg, supply chain, staff time)
• Somewhat easy and convenient for clients	• Provider bias (eg, reluctance to offer it to young women)
• Amenable to community-based distribution	• Poor continuation
	• Implicated for possible increased HIV risk

a Weight gain may be perceived as positive by some women.

## PROVISION BY DRUG SHOPS

A number of studies have previously shown that community health workers can provide injectables safely and effectively.^2^ Thus, it should come as no great surprise, as described in the paper by Akol and colleagues,^3^ that the same is true when drug shops in peri-urban areas of Uganda are properly enlisted to provide them. In fact, a technical consultation in 2013 concluded that, with appropriate training and monitoring, drug shop operators can screen and counsel clients for DMPA effectively and administer DMPA injections safely.^4^ What is actually extremely notable, however, is that in the Akol study the convenience and quality of drug shop provision led the drug shops to reach a substantial share of overall family planning users in their areas, apparently mostly injectables users.

## VIRTUES OF A 6-MONTH INJECTABLE

One potential major improvement would be a 6-month injectable, which has long been an objective of contraceptive developers. As described by McKenna and colleagues,^5^ in this issue of *Global Health: Science and Practice*, renewed efforts are underway to develop such a 6-month product. Using qualitative methods, the authors explore the potential acceptability and program considerations for one. Perhaps not surprisingly, they find a strong interest among providers, policy makers, and program implementers. Moreover, their findings emphasize the issues involved in introduction of even such a minor change in technology, such as regulatory approval, training, and supply chain adjustments. Interestingly, many providers were not aware that the current 3-month DMPA–IM allows for a 1-month “grace period,” during which clients can receive a reinjection. That indicates we need to work harder to achieve better client satisfaction and continuation even with the current DMPA injectable.

## SUBCUTANEOUS DMPA APPEARS BETTER THAN THE CURRENT INTRAMUSCULAR APPROACH

How might such a 6-month approach be possible? Readers may be aware that a new approach to the 3-month DMPA is provided by Sayana Press, which is administered subcutaneously via the Uniject one-time-use injection system. Its proprietary DMPA formulation provides a lower hormonal dose (104 mg vs 150 mg), resulting in lower but still highly effective MPA blood levels without such a high initial peak that is seen with DMPA–IM. Notably, based on studies of the impact on ovarian activity, it appears likely that Sayana Press itself, despite the lower dose, is actually effective for 4 months with a 1-month grace period.^6^ Unfortunately, a major drawback to Sayana Press is its significantly higher cost.

Fortuitously, it appears that a special formulation of DMPA may not be needed for subcutaneous (SQ) administration. Ironically, the current IM formulation when given subcutaneously provides better, more even, and sustained blood levels of the drug.^6^ So one major approach toward a 6-month injectable is to assess what SQ dosage of the current IM DMPA formulation might be needed to achieve the full 6 months (plus 1-month grace period) duration of action.

The current IM formulation of DMPA when given subcutaneously provides better, more even, and sustained blood levels of the drug.

## MORE CHOICE AND DIVERSITY OF METHODS REMAIN CRUCIAL

Whatever the advances toward a better injectable, it remains clear that practical access to a wide variety of methods remains limited for many women and couples. That is particularly true for the long-acting and permanent methods of implants, IUDs, and sterilization, but it is also true of emergency contraception and the Standard Days Method. The fact that use of implants is growing by leaps and bounds^7^ shows the field is making progress. Continuing to expand access to such a broad variety of methods remains the lynchpin of achieving the Family Planning 2020 goals.^8^ *–Global Health: Science and Practice*
